# A novel palpation–based method for tumor nodule quantification in soft tissue—computational framework and experimental validation

**DOI:** 10.1007/s11517-020-02168-y

**Published:** 2020-04-11

**Authors:** Javier Palacio-Torralba, Robert L. Reuben, Yuhang Chen

**Affiliations:** Institute of Mechanical, Process and Energy Engineering, School of Engineering and Physical Sciences, Heriot-Watt University, Edinburgh, EH14 4AS UK

**Keywords:** Tissue mechanics, Quantitative diagnosis, Prostate cancer, Tumor detection

## Abstract

Variation in mechanical properties is a useful marker for cancer in soft tissue and has been used in clinical diagnosis for centuries. However, to develop such methods as instrumented palpation, there remain challenges in using the mechanical response during palpation to quantify tumor load. This study proposes a computational framework of identification and quantification of cancerous nodules in soft tissue without a priori knowledge of its geometry, size, and depth. The methodology, using prostate tissue as an exemplar, is based on instrumented palpation performed at positions with various indentation depths over the surface of the relevant structure (in this case, the prostate gland). The profile of force feedback results is then compared with the benchmark in silico models to estimate the size and depth of the cancerous nodule. The methodology is first demonstrated using computational models and then validated using tissue-mimicking gelatin phantoms, where the depth and volume of the tumor nodule is estimated with good accuracy. The proposed framework is capable of quantifying a tumor nodule in soft tissue without a priori information about its geometry, thus presenting great promise in clinical palpation diagnosis for a wide variety of solid tumors including breast and prostate cancer.

**Electronic supplementary material:**

The online version of this article (10.1007/s11517-020-02168-y) contains supplementary material, which is available to authorized users.

**Graphical abstract:**
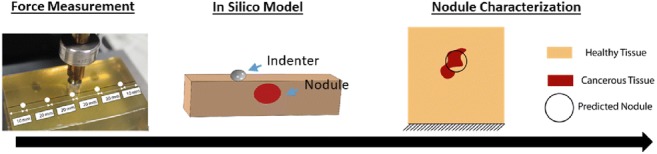
This study proposes a computational framework of quantification of cancerous nodules in soft tissue. The methodology is based on instrumental palpation performed at positions with various indentation depths. The profile of force feedback results is then compared with the benchmark in silico models to estimate the size and depth of the cancerous nodule.

## Introduction

Determining the size and location of a tumor nodule in soft tissue is critical. Ideally, this should occur at the earliest possible stage, giving the best chance of determining the optimal treatment thus improving patient outcomes. However, characterizing cancer, even with the aid of conventional biopsy remains a non-trivial task, particularly for primary diagnosis. Imaging techniques such as magnetic resonance imaging (MRI) have proven useful in detecting pathological conditions such as neoplasms [1], and sonoelastography has also been widely used in clinical diagnosis for such tissues as breast [2] and liver [3]. Although such techniques allow maps of relative tissue density or stiffness, diagnosis still remains challenging in certain scenarios such as co-occurrence of benign prostate hyperplasia [4] or tumors located deeply from a measurable surface [5].

Although prostate cancer may not initially cause symptoms, in later stages, it can lead to difficulty in urinating or pelvic pain and, once it has metastatically spread to places including the lymph nodes [6] or bones, may significantly worsen the prognosis of the patient. The current methods used to diagnose prostate cancer include examination of prostate-specific antigen (PSA) level in blood, digital rectal examination (DRE), biopsies, and various imaging techniques, including multi-parametric magnetic resonance imaging (mpMRI) and trans-rectal ultrasound. These methods vary in their sensitivity and specificity, and limitations include low sensitivity in diagnosis using PSA level alone [7, 8], false negatives [6, 9, 10], and the risk of infection [11] in using biopsies. DRE is a primary diagnostic technique where a clinician’s finger is applied to the palpable surface of the prostate through the rectal wall, looking for abnormalities such as changes in roughness and lumps. It is simple, relatively non-intrusive, and requires no equipment. However, it remains a qualitative diagnostic method that largely relies on the practitioner’s experience and is therefore subjective.

To overcome these limitations, a range of instrumented palpation techniques have been proposed to use quantitative analysis of mechanical measurements of the prostate to improve sensitivity [12, 13]. Instrumented palpation devices, often utilizing automated algorithms through mechanical means such as using rolling, sweeping, or indentation probes [14–16], have been developed to measure tissue elasticity and assess the presence of any abnormality. In addition, autonomous robotic systems have been used to locate tumor nodules in soft tissue and have been applied to ultrasound elastography [17]. Nevertheless, the effects of the depth and size of a nodule in the force feedback during palpation are often coupled. A small nodule near the surface of the tissue and a larger nodule deeper inside the tissue could result in the same local force feedback. Decoupling these effects for the purpose of tumor nodule quantification is of critical importance since the depth, size, and location of a tumor can, in many cases, be related to the progression of the disease [18]. Furthermore, knowledge of the location and size of a tumor nodule can affect the required treatment and the surgical margin, both of which influence the surgical risks and outcome. In the literature, there have been various studies of palpation-based quantitative tumor identification, such as those using computational models with simple geometries of cancerous nodules of cylindrical [19, 20] or rectangular [14] shape, and others based on inverse or optimization methods, such as artificial neural networks, to predict not only the size and depth of anomalies but also their mechanical properties [20]. However, most methods rely on a priori knowledge of the stress distribution in the tissue under certain loading conditions, which remains impracticable in primary diagnosis.

Thus, there is a need of an inexpensive, fast, and reliable method to detect and characterize the cancerous nodules in soft tissue, at the very least to supplement and inform early stage diagnosis. For prostate cancer, this approach would be particularly useful when patient surveillance is required and as a complement to PSA testing and biopsies. To that end, this study presents a novel diagnostic framework using palpation to determine both the size and depth of an anomaly without a priori knowledge of its location or geometry, providing a relatively non-invasive, quantitative tool for early detection, and characterization of tumor nodules.

## Materials and methods

### The computational models

#### In silico configuration

**Fig. 1:**
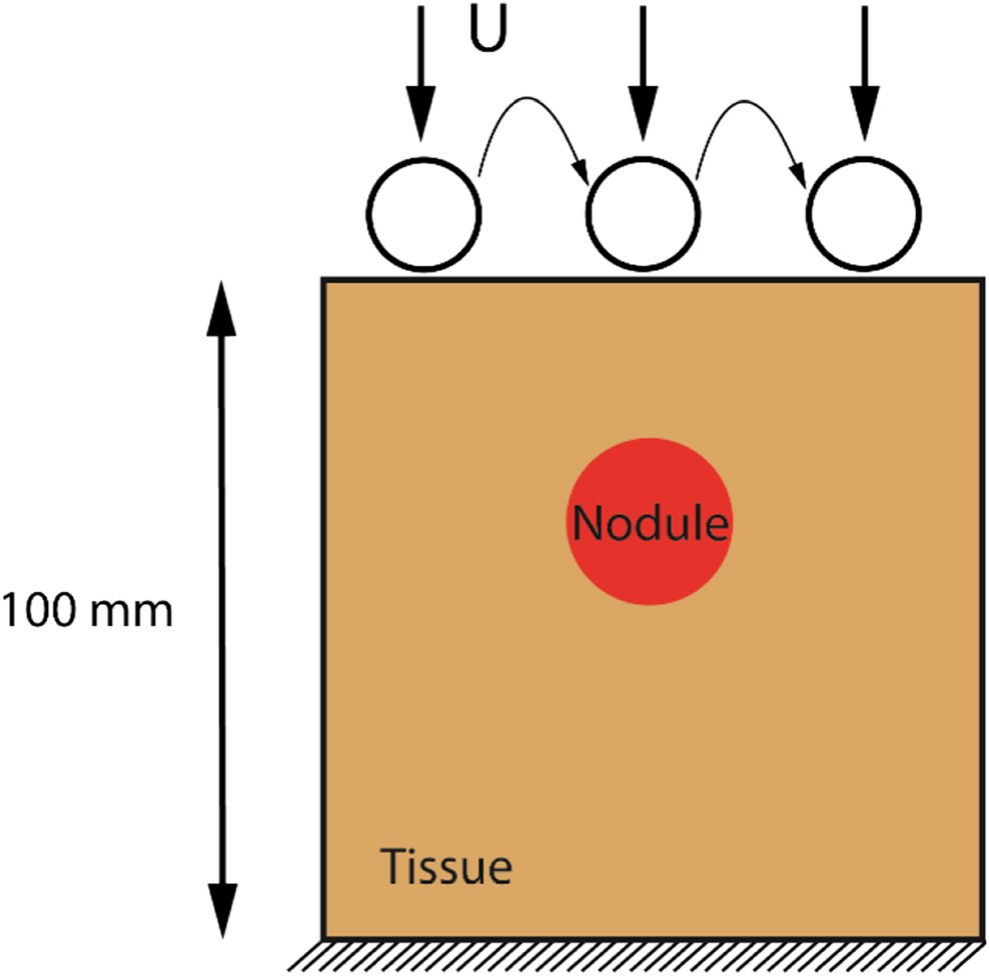
Schematic diagram of the 2D computational model. U denotes the indentation applied with a certain indentation depth, over a number of indentation locations

A 2D *in silico* model was used to demonstrate the feasibility of the proposed methodology. As shown in Fig. 1, it consists of a square domain (100 mm × 100 mm) with a tumor nodule located inside. Instrumented palpation was performed at the upper surface at 40 equally spaced locations, using indentation depths of 1, 5, and 10 mm. It is worth pointing out here that the indentation parameters (depth, number and direction) may be limited by clinical factors such as the acceptable duration of examination, patient comfort, and accessibility from within the rectum. Therefore, a balance needs to be found between detectability and practicability, for which a sensitivity analysis using the model is very helpful, provided that the model can be validated. A cylindrical probe with a spherical tip of diameter 10 mm was used here on the basis that such a probe has already been demonstrated for detecting tumors in soft tissues [19]. The probe was considered to be a rigid body with contact between it and the tissue frictionless [14]. The bottom of the model was constrained to represent the configuration of an ex vivo measurement where the tissue sample lies on a flat testing platform [20, 21]. The “test” consisted of applying a quasi-static vertical displacement and recording the force feedback.

#### Material model and parameters

As with most biological tissues, prostate tissue exhibits a certain amount of viscoelasticity [13, 22] and its mechanical behavior may depend on the strain/loading rate [12, 13]. However, the approach adopted here is to make use of the (quasi-)elastic behaviors of the materials and prostatic tissue under low deformation rates. In such cases, the viscous response of the materials becomes negligible and this approach has been widely used in the literature [21, 23]. To satisfy this, indentations are conducted using a low indentation rate (0.1 mm/s) and allowing sufficient waiting time (60s) at the desired indentation depth (see below).

Experimentally measuring the elastic properties of the prostate tissue and their cancerous counterparts is not trivial. This is due to the nature of prostate tissue being glandular tissue (filled with prostatic fluid); therefore, it is not ideal to utilize commonly used approaches of soft tissue measurements where tissue is cut into, for example, thin sheets or cylindrical cores, for mechanical characterization. Table 1 summarizes a selection of published values for the elastic properties of prostate tissue, revealing a wide range attributable to inter-patient variations (within investigator) and different experimental configurations (between investigators). In the interest of focusing on detectability, for our study, non-cancerous and cancerous tissues are modeled as incompressible elastic materials, with equivalent Young’s moduli of 20 kPa and 40 kPa, respectively, adopted from Hoyt et al. [13].

**Table 1:** Mechanical properties of the prostate tissue, including healthy and cancerous ones, reported in the literature

Young’s modulus (kPa) Healthy tissue	Young’s modulus (kPa) Cancerous tissue	Poisson’s ratio	Reference
12	200	0.499	[14]
30	90	0.495	[22]
16 ± 5.7	40 ± 15.9	0.49–0.5	[13]
3	19		[15]
41	135		[12]
10~29	11–38		[23]

To overcome potential numerical issues at high strain range caused by the deep indentation while maintaining a sufficient accuracy, the elastic moduli of both tissues are fitted with the second-order hyperelastic Ogden strain energy density function, as

1 $$ \Psi =\sum \limits_{i=1}^2\frac{2{\mu}_i}{\alpha_i^2}\cdotp \left({\overline{\lambda_1}}^{\alpha_i}+{\overline{\lambda_2}}^{\alpha_i}+{\overline{\lambda_3}}^{\alpha_i}\right)+\sum \limits_{i=1}^2\frac{1}{D_i}\cdotp {\left({J}_{el}-1\right)}^{2i} $$

where μ_i_ and α_i_ are the material parameters to be fitted and $\overline{\uplambda_1},\overline{\uplambda_2},\overline{\uplambda_3}$ are the principal stretches. Finite strains were considered in the model, which was solved using the finite element method in ABAQUS (Dassault Systemes, Vlizy-Villacoublay, France). The resulting parameters used for the Ogden model in ABAQUS, fitted against the elastic moduli as mentioned above using Eq. (1), are shown in Table 2.

**Table 2:** Parameters of the second order Ogden strain energy density model for the healthy prostate tissue and the cancerous nodule, respectively

Tissue	*μ*_1_	*α*_1_	*μ*_2_	*α*_2_	*D*_1_	*D*_2_
Prostate	0.02119	2.244	− 0.01120	− 1.081	0	0
Cancerous nodule	0.04238	2.244	− 0.02240	− 1.081	0	0

#### Sensitivity analysis—influence of nodule geometry

**Fig. 2:**
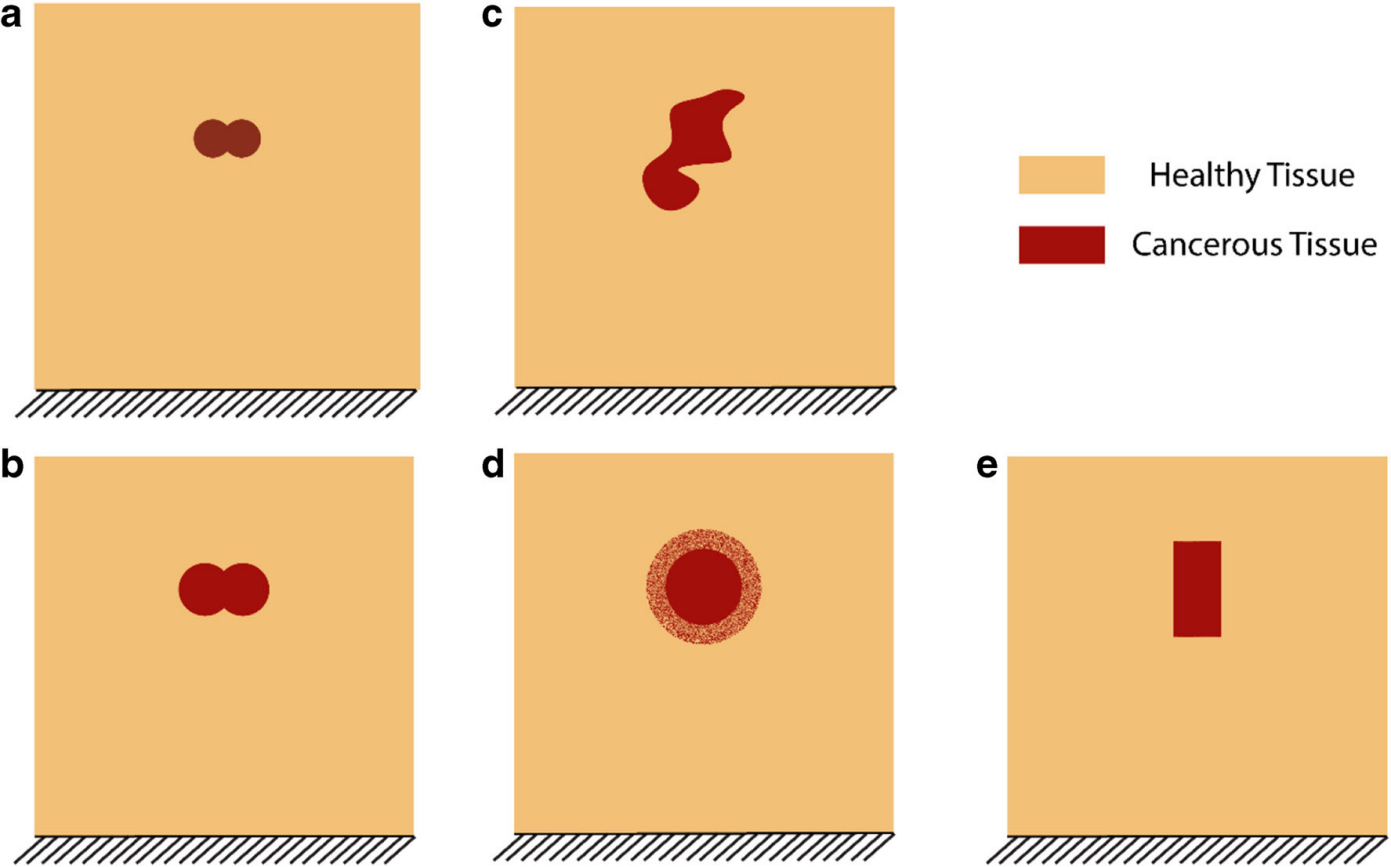
Different scenarios of cancerous nodules considered to test the effectiveness of the proposed methodology. **a**, **b** are built from intersecting circle in different sizes (10 mm and 15 mm in diameter, respectively). **c** Tumor nodule is in a arbitrary shape. **d** Circular nodule surrounded by a mixture of both tissues (20 mm inner diameter and 30 mm outer diameter). **e** Nodule in a rectangular shape (25 mm in height and 12.5 in width)

To demonstrate the capability of the proposed method, four different nodule geometries were considered for a total of five different cases. The first two cases comprised two intersecting circular nodules of different diameters, as shown in Fig. 2a, b. This represents scenarios where a tumor is growing from two different locations or growing around the urethra [24]. A tumor of arbitrary shape was also considered, Fig. 2c and one with an interfacial layer between the cancerous nodule and the healthy tissue (Fig. 2d), where the nodule is surrounded by a mixture of healthy and cancerous tissues. Such a scenario is often found in clinical cases, where it is difficult to draw a clear boundary between healthy and cancerous tissue without local histopathological examination. Finally, a nodule of rectangular shape was included (Fig. 2e) for comparison with the experimental validation model, described later.

### The computational framework—decoupling the size and depth of tumor nodule

As mentioned above, the size and depth of a nodule are often coupled in the force feedback from instrumented palpation. The methodology proposed to overcome this consists of two stages: nodule localization and nodule quantification. It has been suggested [14, 19] that the location of a nodule can be determined by finding the point where the difference between the force feedback and that from the “healthy” background reaches a maximum. Such a procedure only requires the force feedback to be recorded at a single depth of indentation, which is not sufficient to determine both size and depth of a nodule, as the size and depth can both affect the force feedback of indentation measurement in a similar way, leading to potential ambiguity. Although the force feedback curves for a deeply embedded nodule and a healthy sample are similar, they become progressively different (different curvature of the force-displacement profile) as the nodule is closer to the surface of indentation. It is hypothesized, and later validated, that such variations in the curvature of the force feedback profile can be affected in distinct ways by size and depth of the nodule, based on which the quantification of the tumor nodule is performed.

**Fig. 3:**
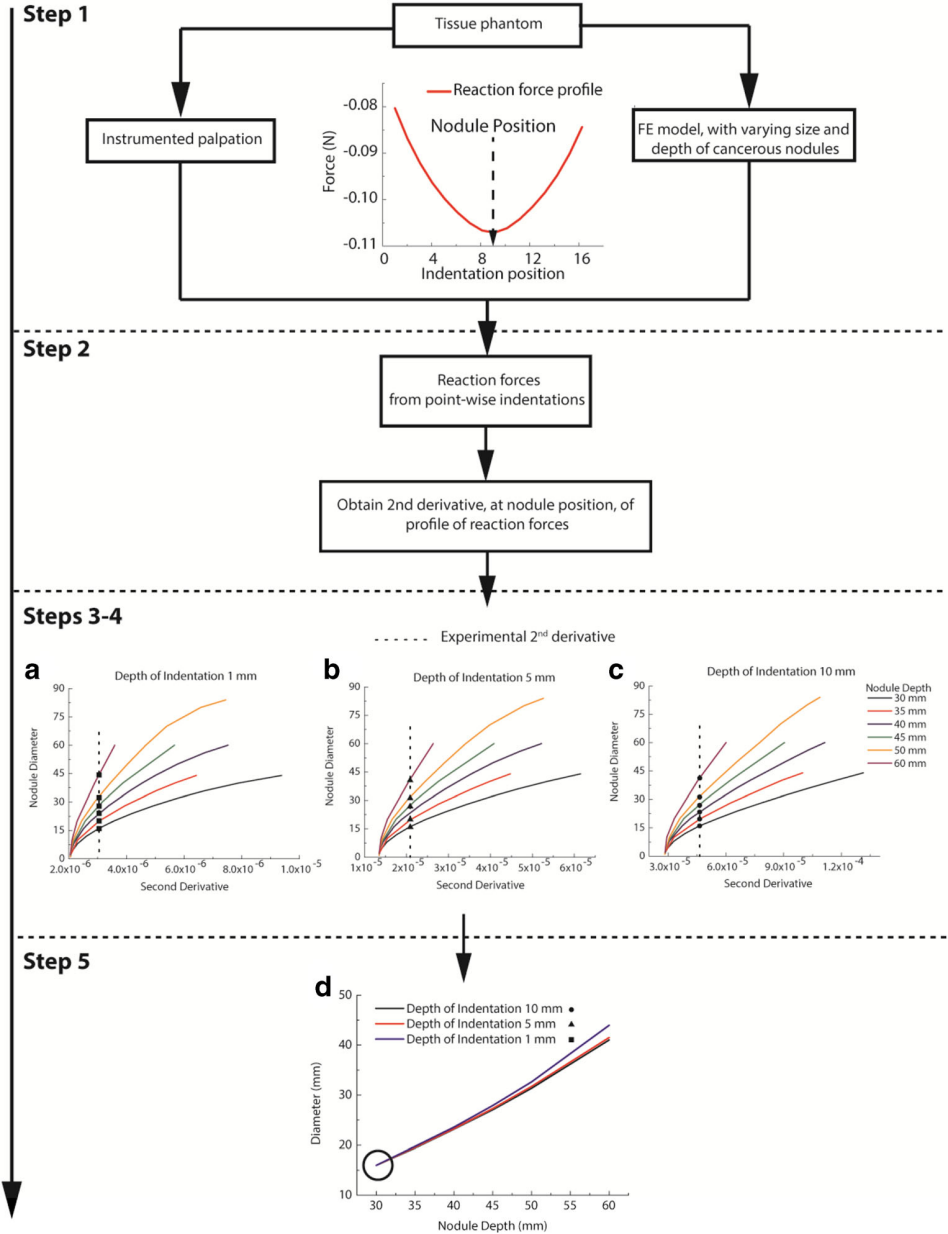
The proposed schema for developing a localization/quantification/calibration process. Step 1: Acquire reaction force data from point-wise indentations at different depths of indentation across the surface. Step 2: obtain the second derivatives of the force feedback profiles. Step 3: the second derivative at the nodule position is then plotted against the nodule diameter, based on the FE results, for each indentation depth. Step 4: the 2nd derivatives obtained from the experimental measurements for each indentation depth are plotted on top of those from Step 3, leading to a number of intersecting points, represented by the symbols black square, black triangle, and black circle. Step 5: the intersecting points are then plotted in the nodule depth/diameter figure, and a set of curves allows identifying the size and depth of the nodule

The proposed schema for developing a localization/quantification/calibration process is illustrated in Fig. 3, involving five steps:

Step 1: The FE models are constructed based on the dimensions of the tissue phantom. The cancerous nodule in the FE models has varying size and depth; however, in the experiment, there is only one set of values for size and depth of the cancerous nodule. Point-wise indentations are conducted at the surface of the phantom, both experimentally and computationally, at multiple depths of indentation (e.g., 1, 5, and 10 mm)
Step 2: Obtain a series of line profiles of indentation force for each scenario and each indentation depth. Obtain the second derivative (with respect to indentation position) of the force feedback profile using a smooth spline over all data points
Step 3: Plot the value of the second derivative at the position where the tumor is located against the diameter of the nodule (known in the FE models) for different indentation depths
Step 4: Plot the value of the second derivative at the tumor location from the experimental measurement on top of the data derived from in silico models from Step (3) to generate a set of intersecting points of depth and diameter, for each indentation depth used
Step 5: Plot the intersecting points on a depth-diameter diagram representing the correlation between datasets to indicate the depth and size of the nodule. For nodules with irregular shapes, the estimated equivalent radius is defined as

2 $$ {R}_{equivalent}=\sqrt[3]{\frac{3\cdotp {V}_{nodule}}{4\cdotp \pi }} $$

where *V*_*nodule*_ denotes the volume of the nodule.

### Experimental validation

#### Material characterization of gelatin phantom

A gelatin phantom which mechanically mimics the prostatic tissue was used to validate the proposed framework. Such material has been widely used for surgical training [25] and ultrasound diagnosis [26] for prostate. To make the phantom materials, gelatin powder was mixed with boiling water (144 g/l for “cancerous” and 120 g/l for ‘healthy’ samples) and the cancerous sample was dyed using the red food colorant. The mixture was then slowly stirred to avoid bubble formation until it became homogeneous and transparent. After cooling to room temperature, the samples were stored at 4 °C for 18–20 h. To make the tissue phantom, a block 100 mm × 31 mm × 60 mm was prepared consisting of healthy gelatin with an embedded tumor nodule of dimensions 20 mm × 12 mm × 12 mm. This was achieved using a first layer of healthy gelatin onto which the pre-solidified cancerous nodule was placed before it had completely set. The remaining healthy gelatin was then poured in so that the top surface of the cancerous nodule was located 10 mm below the surface of the sample. Such a configuration was reasonably representative of the clinical situation as tumors are often found near the posterior surface of the prostate [27]. Before testing, the tissue phantom was given 90 min to reach the room temperature. It should be noted here that the shape of tumor nodule was idealized in order that it could be made and measured more accurately and be used to validate the computational model similar to Fig. 2e.

The indentation measurements, in line with the proposed modeling methodology, aimed to measure the elastic response, in the form of force measurements, at the desired indentation depth. To achieve that, careful considerations were given to the design of the experimental protocols, mainly to ensure consistency among all indentation measurements and to minimize the potential influence of viscoelastic behaviors of gelatin materials, including (i) the indentation rate/speed was set to be 0.1 mm/s; (ii) before the force data was taken for each probing point, sufficient time was allowed for the phantom materials to relax (60 s) and the sign of stress relaxation was no longer observed; (iii) between two consecutive probing, sufficient time was given to make sure the first probing point showed no observable sign of indentation deformation; and (iv) spacing of 20 mm was given between two adjacent probing points on the phantom.

For the benchmarking process, two different material phantoms were prepared: a healthy one consisting entirely of the softer gel and a cancerous one, consisting entirely of the stiffer gel. The material phantoms were characterized using a total of 9 indentation measurements, three different indentation depths (i.e., 2/4/6 mm) at three locations (considering the symmetry of the sample). The same configuration (including the depth and location of indentations and the material geometries) was modeled using finite element in ABAQUS, using the incompressible neo-Hookean model:

3 $$ \Psi ={C}_1\left(\overline{I}-3\right) $$

where $\overline{I}$ is the first invariant of the left Cauchy-Green tensor and C_1_ the material constant. The material phantoms were mechanically characterized in such a way that the difference in the force feedback between the experimental measurement and finite element simulations was minimized using Levenberg-Marquardt’s algorithm [28], a damped method commonly used to perform nonlinear least square approximations:

4 $$ \mathit{\min}\sum \limits_{depth=1}^n\sum \limits_{pos=1}^m{\left({F}_{FE}\left({C}_1\right)-{F}_{Exp}\right)}^2 $$

where *F*_*FE*_ and *F*_*Exp*_ denote the force feedback data from finite element analysis and experimental measurement, respectively, and *n* denotes the number of indentation depths and m the number of indentation positions used. In this way, the optimal *C*_1_ is found by converging the reaction forces at the indenter considering all force measurements at various indentation depths and positions, and the mechanical properties of the two gelatin materials (i.e., cancerous, red, and non-cancerous, yellow) are then derived.

#### Instrumented palpation on tissue phantom

**Fig. 4:**
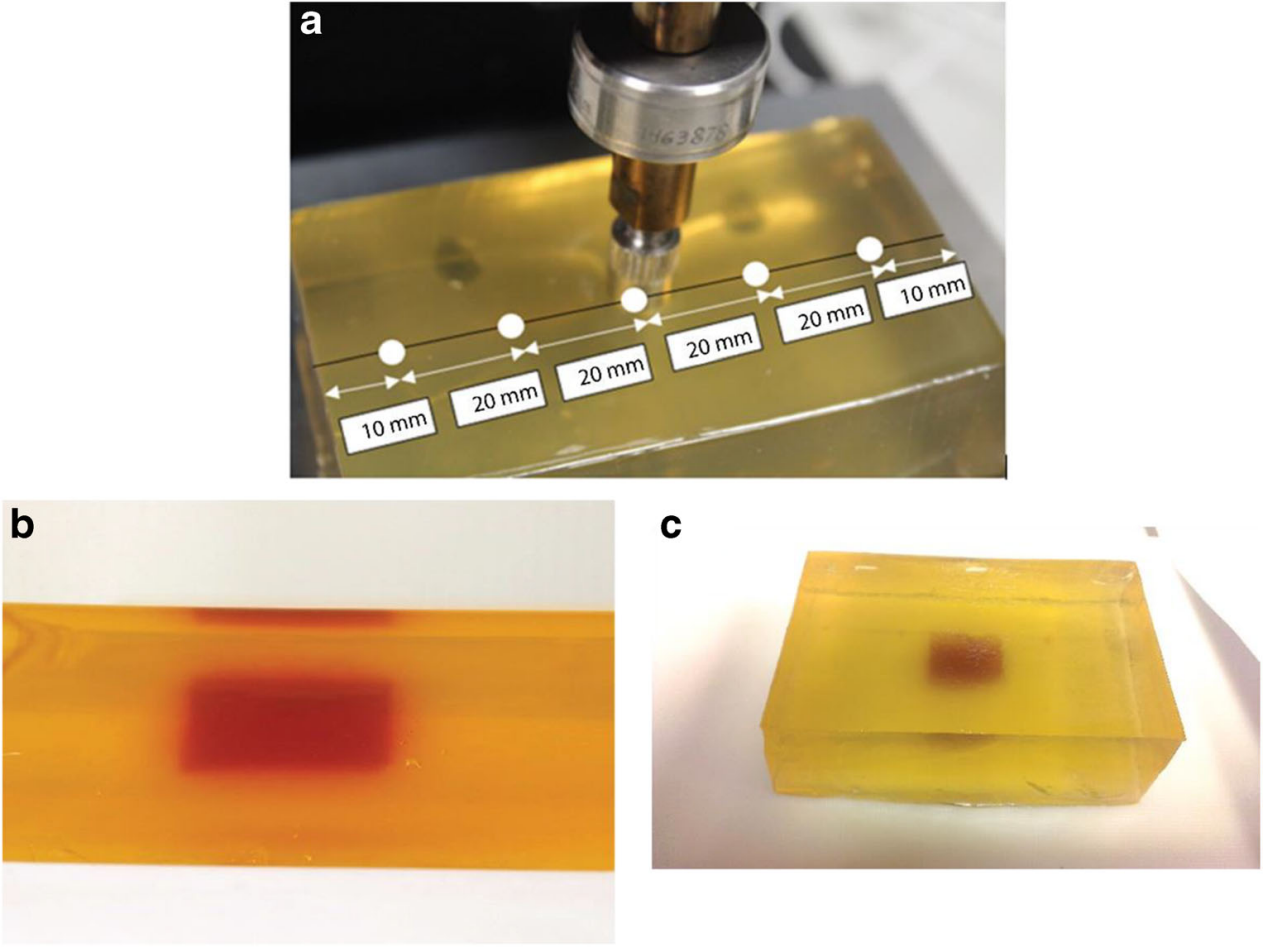
The gelatin tissue phantom and experimental configuration. **a** The positions where indentation is performed. **b**, **c** The nodule (red) inside the phantom (light yellow). The size of gelatin phantom is 100(length) × 31(height) × 60(width) mm and the cancer nodule 20 × 12 × 12 mm at the depth of 10 mm from the top surface of the phantom

The tissue phantom was subjected to point-by-point indentation measurements over its top surface, using indentation test machine, Mach-1 V500css (Biomomentum Inc., Laval, Canada). A 10-mm diameter hemispherical indenter, (as used for computational analysis) was with three different indentation depths (i.e., 2, 4, and 6 mm), respectively. The diameter was chosen to be 10 mm, as a compromise between covering the entire measureable surface with a reasonable number of indentations and having sufficient sensitivity to detect the edges of the nodule. The long-term modulus was determined at 150 s, in order to minimize the effect of any viscoelastic behavior in the gelatin materials. It should be noted here that the number of indentation sites and the depths of indentation were chosen within certain constraints. The indentation sites must have sufficient distance between them to minimize the influence of inelastic effects such as long-term viscoelastic behavior. The smallest indentation depth was chosen to be 2 mm, to ensure an adequate signal to noise ratio, while the largest indentation depth of 6 mm was chosen as a limit beyond which patient discomfort might ensue in a clinical situation. The phantom was visually examined after every indentation test to ensure that no material damage had been caused by the measurements. The probe points are illustrated in Fig. 4a, while Fig. 4b, c shows the location of the cancerous nodule (red) within in the healthy (pale yellow) matrix. The recorded force feedback data of was then processed using the proposed framework to identify and characterize the tumor nodule.

## Results and discussion

### Quantitative identification of cancerous nodules: 2D analysis

This section illustrates, firstly, how using a single indentation depth is insufficient to determine the size and depth of a cancerous nodule, and then demonstrates, by examples, how the proposed methodology quantifies the depth and size of a tumor nodule.

As mentioned above, one of the major challenges in identification of tumor nodules in soft tissue is the ambiguity that arises where a smaller nodule closer to the surface could give similar force feedback to a larger one located deeper inside the tissue. To demonstrate the coupling effect of size and depth, a parametric study was carried out using circular tumors with the maximum size at each depth within the practical constraints for the particular case considered, as shown in the electronic supplementary material ESM-Fig. 1(a)-(b). ESM-Fig. 1(c) shows the “envelope” of maximum force feedback obtained for different combinations of tumor depth and size subject to indentations of depth 1 and 10 mm, respectively. For any given force feedback, the solutions of the nodule depth and size are not unique in that different combinations of the depth and size exist and could lead to the same force feedback. It is therefore impossible to quantify the size and depth of the nodule from palpation measurements using a single indentation depth. This demonstrates a critical challenge in palpation-based diagnosis, especially when the ratio between nodule diameter and depth is small. Put bluntly, large tumors located deep in the prostate (i.e. far away from the rectal wall) could be identified as a smaller, more superficial, benign tumor.

**Fig. 5:**
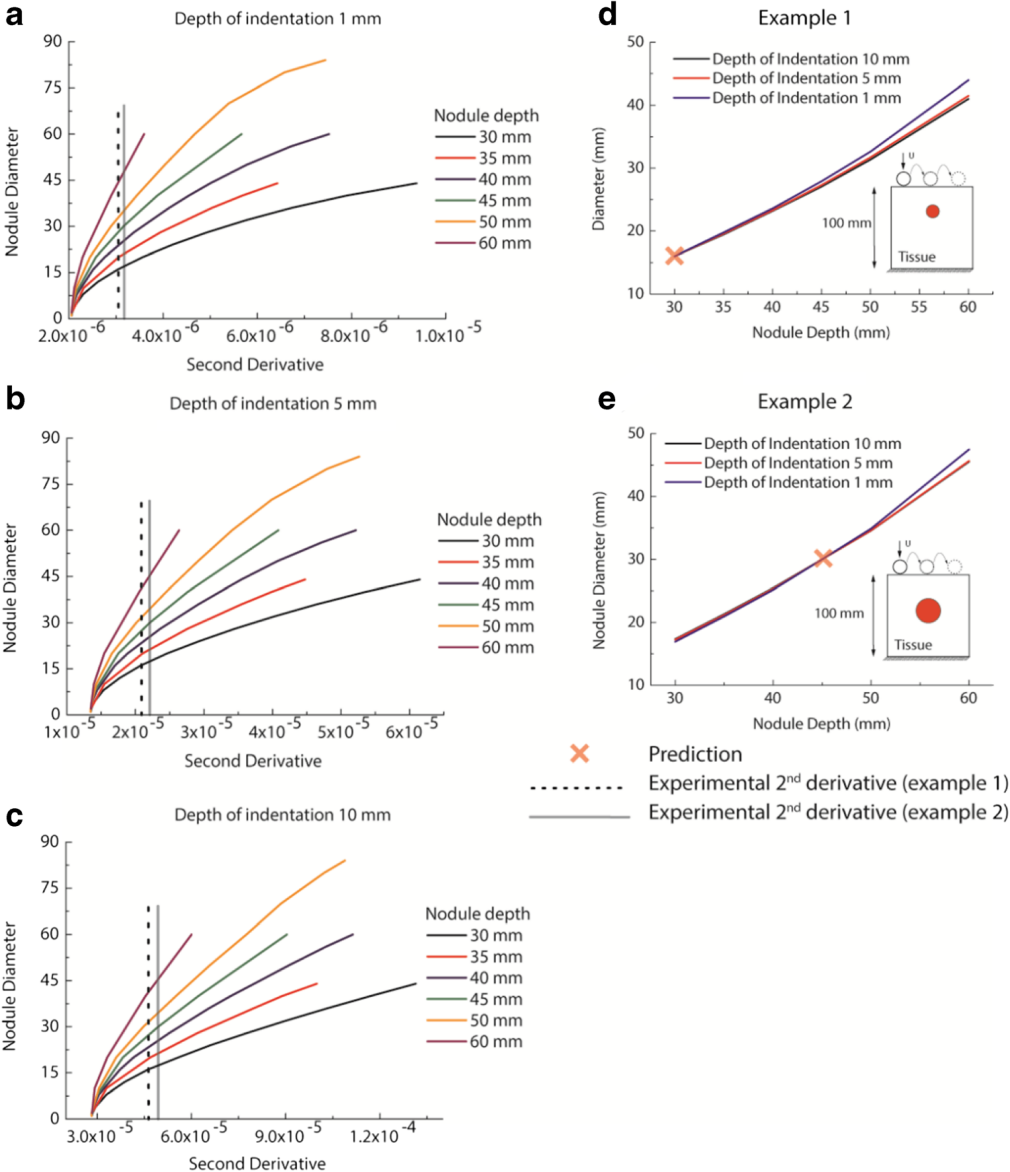
The procedures of quantification of cancerous nodules are explained here, where the nodule has different sizes and depths. The diameter of the cancerous nodule against the second derivative of the force profile at the tumor position is plotted, when using a depth of indentation of 1 mm (**a**), 5 mm (**b**), and 10 mm (**c**). The depths of the tumor, in computational models, are 30, 35, 40, 45, 50, and 60 mm. **d** The point of intersection is (30, 16) in example 1, which indicates the true values of the tumor diameter (16 mm) and depth (30 mm). **e** The point of intersection is (45, 30) in example 2, which indicates the true values of the tumor diameter (30 mm) and depth (45 mm)

Figure 5 shows two examples to demonstrate the procedures of the proposed methodology. In the first example, a circular tumor of 16 mm diameter located at a depth of 30 mm was considered and, in the second one, a 30-mm diameter nodule located at a depth of 45 mm. The second derivatives of the force feedback profiles from the experimental measurements were derived and plotted against the same dataset but calculated from in silico models (i.e., with circles of different sizes located at various depths) as shown in Fig. 5a–c. Following the steps illustrated in Fig. 3, the nodules were identified and quantified as shown in Fig. 5d, e. It can be seen that all depth-size curves intersected at one point which accurately estimated the depth and size of the cancerous nodule.

### Variations in nodule geometry—a sensitivity study

**Fig. 6:**
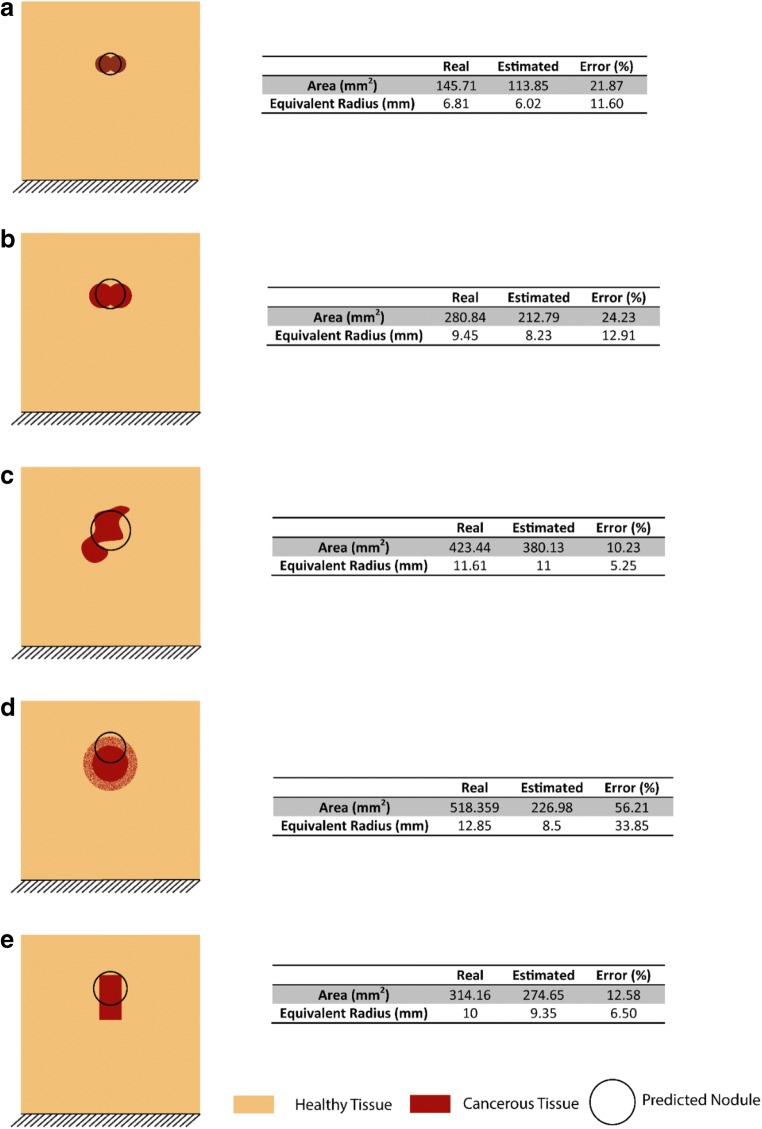
Geometries of 2D tumor nodules used to test the effectiveness of the proposed methodology and the predicted nodule depths and sizes

The proposed methodology was then applied to tumor nodules of different and irregular geometries, in order to further demonstrate the feasibility of the method. Figure 6a and b show the results for the identification of nodules with shapes similar to those found close to the urethra in the prostate. The area was estimated with an error below 25%, and the radius of the equivalent tumor nodule was estimated with an error of 11.6%, while the estimated depth was close to the center of the nodule. Although the cancerous nodules in most examples presented here were of regular shapes, either in rectangular or circular geometries, this enabled a more convenient comparison of the effect of tumor geometry on the quantification outcome. In Fig. 6c, where an irregular nodule was present, the area was estimated with an error of around 10% with a good agreement in nodule depth. Cancerous nodules in prostate are often of irregular shape, similar to the case shown in Fig. 6c. In Fig. 6d, an additional scenario was considered where the tumor boundary was not well defined. The tumor area and depth were both underestimated in this case with relatively high error, due to a higher ambiguity in the force feedback arising from the “transition zone.” It should be noted that, in this case, it is possible that the area that contains a highly diffuse tumor or boundary areas of an un-confined nodule could have different mechanical behavior to the main part of the nodule, and that it may be necessary to introduce a material model that operates beyond the elastic regime, a complication not pursued in the current study. Finally, a nodule of a rectangular shape was considered in Fig. 6e, with an error of 12.58%.

### Experimental validation using gelatin phantom

**Fig. 7:**
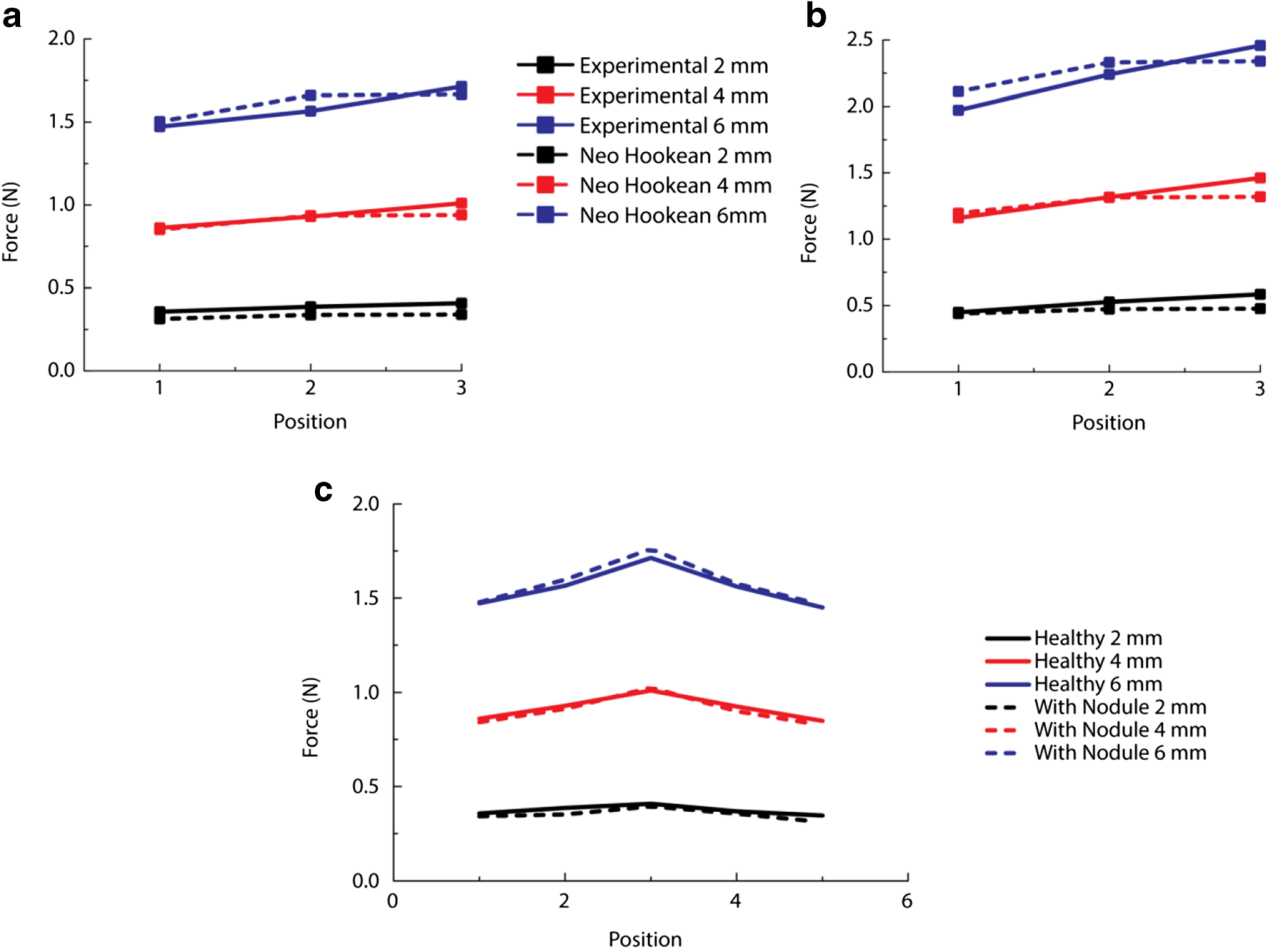
Results of the instrumented palpation on the healthy (**a**) and cancerous (**b**) material phantoms and the fitted neo-Hookean model. **c** Comparison of the healthy sample and the sample with a nodule embedded. Note that the symmetry condition is used in **a**, **b**, where position 3 is the middle point of the phantoms

The proposed computational framework was then validated using data from the experimental measurements with gelatin phantoms. In order to do this, the mechanical properties of the material phantoms need to be characterized first. Figure 7a and b show the force feedback obtained from the experimental material phantoms fitted to the incompressible neo-Hookean model, Eq. (3), optimized using the Levenberg-Marquardt algorithm, which resulted in values for the Young’s modulus of 28.1 kPa and 39.5 kPa for the healthy and cancerous tissues, respectively. Table 3 shows the errors in estimating the material parameters using the neo-Hookean model. It should be noted that the maximum fitting errors (16.7% and 18.45% for healthy and cancer, respectively) occurred in both cases at the mid-point of the sample when the smallest depth of indentation was used.

**Table 3:** Results of fitted material properties for the healthy and cancerous material phantoms, respectively

	C_1_	Max error	Min error	Mean error	E (kPa)
Healthy	4.6898e−3	16.7%	0.6%	6.82%	28.1
Cancer	6.5872e−3	18.45%	0.22%	6.65%	39.5

The force feedback for palpation of the tissue phantom (containing the cancerous nodule of size 20 × 12 × 12 mm at a depth of 10 mm) is shown in Fig. 7c and compared with the fully healthy sample, illustrating the more “peaky” nature of the scan (larger second derivative) when a tumor was present. Whereas the absolute value of the feedback force was generally higher at higher indentation depths, it must be noted that the reverse was the case for the 2-mm indentation. This can be attributed to the difficulties in robustly finding the contact in the experimental indentation, less noticeable for deeper indentation depths where the force feedback was significantly higher than the “noise” recorded during the contact-finding process. This would be of critical importance in clinical applications, especially during surgery (e.g., nodule identification prior to tissue removal) when blood clots or other debris, along with working access constraints, may lead to complications in achieving a robust detection of contact.

**Fig. 8:**
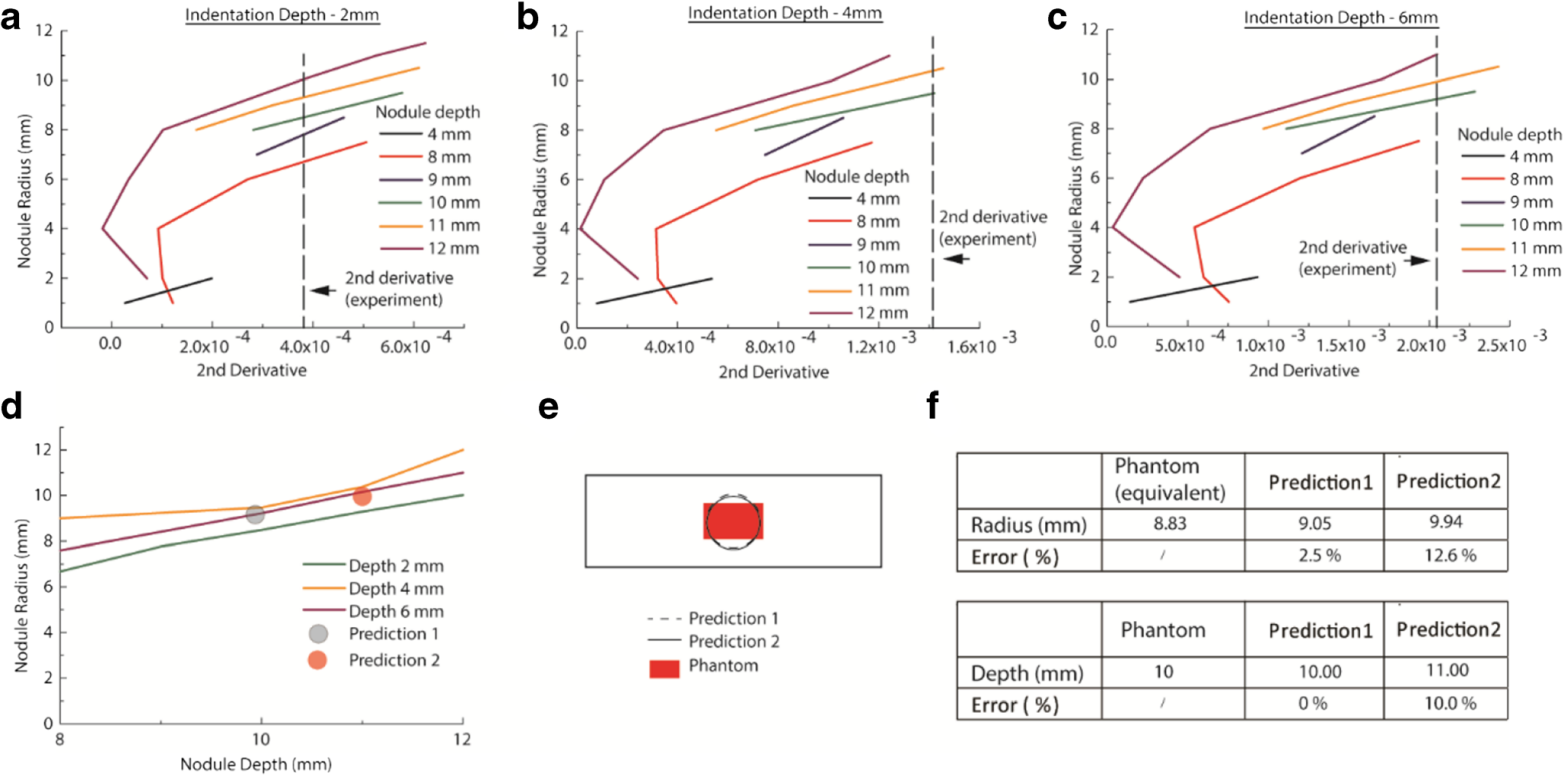
Experimental validation of the proposed method using the gelatin phantom. **a**–**c** Results of the second derivative of the force feedback profile obtained from computational models where nodules of different sizes are located at various depths (4, 8, 9, 10, 11, and 12 mm). The vertical dashed lines indicate the value of 2nd derivative obtained from experimental measurements. **d**, **e** Results of nodule depth and size identification. **f** Error between the phantom and predicted parameters

Figure 8 summarizes the experimental validation of the method designed to overcome these limitations. After the material phantoms were characterized, point-by-point indentations were carried out on the tissue phantom, as illustrated in Fig. 4. Experimental data of force feedback, i.e., indentation over 5 locations using indentation depths of 2/4/6 mm, were obtained and then put through the framework demonstrated in Fig. 2. Similarly, multiple *in silico* models were also run with spherical cancerous nodules of different diameters located at various depths (i.e., 4, 8, 9, 10, 11 and 12 mm). The second derivatives of the force feedback profiles from the *in silico* models were then plotted in Fig. 8a–c for indentation depths of 2, 4, and 6 mm, respectively. It should be noted here that it is possible to further increase the number of nodule depths used in *in silico* models, although this would also lead to higher computational cost. As it was, the datasets in the depth-radius plots, i.e., Fig 8d, did not intersect at a single point, possibly due to experimental errors in the indentation measurements. For this example, two possible solutions, highlighted in circles, were obtained where the three datasets approach each other most closely; Fig. 8e shows the corresponding quantification errors. The chosen solution (prediction 1, highlighted in gray) led to an estimated radius of 9.05 mm, compared to the equivalent radius of the cancerous nodule in the tissue phantom of 8.83 mm, an error of 2.5%. The depth of the nodule was predicted to be 10 mm, the same as the true depth of the nodule in the phantom. If the other solution (i.e., red/prediction 2) was chosen, the estimated radius became 9.94 mm (i.e. an error of 12.6%) and the depth 11 mm (i.e. an error of 10%).

It is important to note here that, in practical uses of the proposed method based on the force measurements obtained experimentally, the error in estimating the tumor volume could arise through a number of factors, including (i) the inaccuracy in finding the contact point in experimental measurement leads to a certain error in measuring indentation depth. This would be a particular problem for indentations of smaller depth, less than around 1 mm, and it is for this reason that the indentation depth used for the experiments was kept higher than 1 mm to ensure good accuracy in finding the contact point before indentation measurement; (ii) the friction coefficient between the indenter and the tissue is hard to measure. In the in silico model, the contact was considered frictionless; however, it has been shown that friction between the indenter and the tissue may influence the force feedback in experiments [29]; (iii) compared to previous work in nodule characterization [14, 19, 20], the ratio between the cancerous and healthy stiffness considered in this study was rather low, thus making the this study even more challenging.

## Concluding remarks

There is a need for a quantitative strategy in determining the depth and size of cancerous nodules in soft tissue, e.g., prostate, using instrumented palpation for clinical application of nodule identification and characterization. A novel methodology based on mechanical probing and finite element analysis to quantify the size and depth of nodules without a priori knowledge of the topology of nodule and/or stress distribution in the tissue was proposed in this study. Gelatin phantoms with tissue-mimicking mechanical properties were then used to validate the proposed methodology, where both the size and depth of the nodule were estimated with good levels of accuracy, therefore making it a useful complimentary tool for characterizing a variety of tissues such as breast, prostate, liver, and kidney for the purpose of tumor detection and robot-assisted surgery [30].

This study, as it currently stands, has a number of limitations. Firstly, it is worth noting that the materials in this study were considered to be elastic, due to the indentation measurements being conducted with a low indentation rate and long waiting time at the desired indentation depth. Viscoelastic behavior of examined tissue and how it could affect the identification outcome will be studied in future work. Secondly, the ratio of moduli between cancerous and non-cancerous tissues in this study was 2:1, which was somewhat a conservative estimation, compared to findings from many other studies (see Table 1). Should such a ratio be higher than 2:1, one would expect the proposed methodology of cancer nodule detection to be even more sensitive. This will be investigated in detail in a future study, where the prostate tissue phantoms could also be tuned to reflect on different ratios of moduli between cancerous and non-cancerous phantoms. Finally, future studies will also be carried out to further test and validate the proposed method for in vivo applications, potentially with multiple tumor nodules and heterogeneity [31] in the tissue.

## Electronic supplementary material

ESM 1 (DOCX 140 kb)
