# Supplementary material for: A novel palpation–based method for tumor nodule quantification in soft tissue—computational framework and experimental validation
Source: Med Biol Eng Comput. 2020 Apr 11;58(6):1369–81. doi: 10.1007/s11517-020-02168-y (PMC7211792; doi:10.1007/s11517-020-02168-y)
Supplement: Supplementary file 1 — (DOCX 140 kb) [file 11517_2020_2168_MOESM1_ESM.docx]

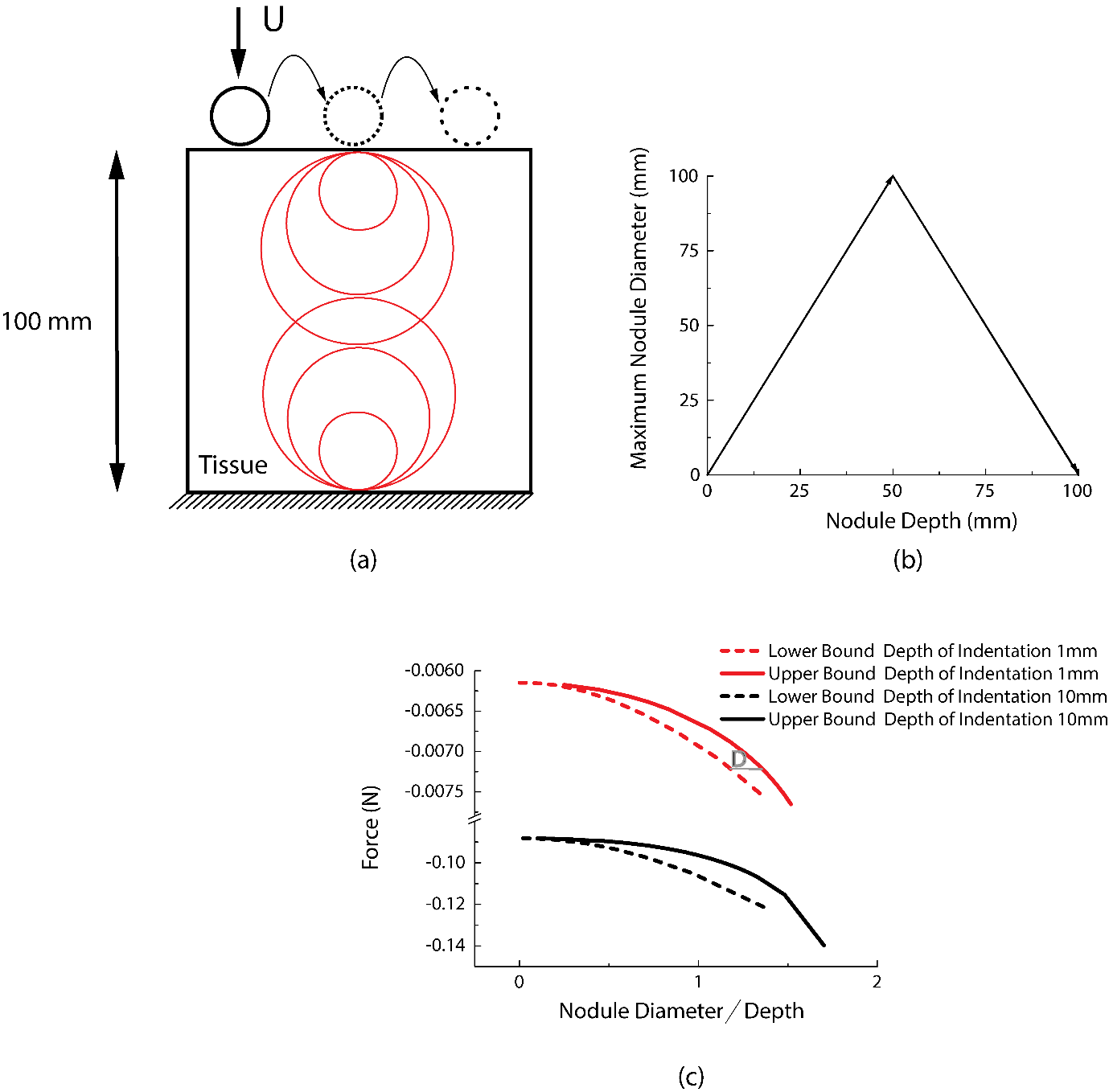


Electronic Supplementary Material (ESM)-Figure 1. The limitations of palpation-based diagnosis using a single indentation depth are illustrated here. (a)-(b) possible combinations of nodule depth and sizes are represented; (c) The envelope of probing forces using a depth of indentation of 1 mm or 10 mm is shown here. For any given force a number of possible combinations of nodule size and depth exist, e.g. segment D.
